# Neuropathological criteria of anti-IgLON5-related tauopathy

**DOI:** 10.1007/s00401-016-1591-8

**Published:** 2016-06-29

**Authors:** Ellen Gelpi, Romana Höftberger, Francesc Graus, Helen Ling, Janice L. Holton, Timothy Dawson, Mara Popovic, Janja Pretnar-Oblak, Birgit Högl, Erich Schmutzhard, Werner Poewe, Gerda Ricken, Joan Santamaria, Josep Dalmau, Herbert Budka, Tamas Revesz, Gabor G. Kovacs

**Affiliations:** 1 Neurological Tissue Bank of the Biobanc-Hospital Clinic, Institut d’Investigacions Biomediques August Pi i Sunyer (IDIBAPS), Barcelona, Spain; 2 Institute of Neurology, Medical University of Vienna, Vienna, Austria; 3 Neurology Department, Hospital Clinic de Barcelona, IDIBAPS, Barcelona, Spain; 4 Neuroimmunology Program, Institut d’Investigacions Biomèdiques August Pi i Sunyer (IDIBAPS), Barcelona, Spain; 5 Queen Square Brain Bank for Neurological Disorders, UCL-Institute of Neurology, University College London, London, UK; 6 Neuropathology, Lancashire Teaching Hospitals NHS Foundation Trust, Preston, UK; 7 Institute of Pathology, Faculty of Medicine, University of Ljubljana, Ljubljana, Slovenia; 8 Department for Vascular Neurology and Intensive Neurological Therapy, University Medical Centre Ljubljana, Ljubljana, Slovenia; 9 Department of Neurology, Medical University of Innsbruck, Innsbruck, Austria; 10 Department of Neurology, University of Pennsylvania, Philadelphia, PA USA; 11 Institució Catalana de Recerca i Estudis Avançats (ICREA), Barcelona, Spain; 12 Institute of Neuropathology, University Hospital Zurich, Zürich, Switzerland

**Keywords:** IgLON5, Parasomnia, NREM, Tauopathy, Brainstem

## Abstract

We recently reported a novel neurological syndrome characterized by a unique NREM and REM parasomnia with sleep apnea and stridor, accompanied by bulbar dysfunction and specific association with antibodies against the neuronal cell-adhesion protein IgLON5. All patients had the HLA-DRB1*1001 and HLA-DQB1*0501 alleles. Neuropathological findings in two patients revealed a novel tauopathy restricted to neurons and predominantly involving the hypothalamus and tegmentum of the brainstem. The aim of the current study is to describe the neuropathological features of the anti-IgLON5 syndrome and to provide diagnostic levels of certainty based on the presence of associated clinical and immunological data. The brains of six patients were examined and the features required for the neuropathological diagnosis were established by consensus. Additional clinical and immunological criteria were used to define “definite”, “probable” and “possible” diagnostic categories. The brains of all patients showed remarkably similar features consistent with a neurodegenerative disease with neuronal loss and gliosis and absence of inflammatory infiltrates. The most relevant finding was the neuronal accumulation of hyperphosphorylated tau composed of both three-repeat (3R) and four-repeat (4R) tau isoforms, preferentially involving the hypothalamus, and more severely the tegmental nuclei of the brainstem with a cranio-caudal gradient of severity until the upper cervical cord. A “definite” diagnosis of anti-IgLON5-related tauopathy is established when these neuropathological features are present along with the detection of serum or CSF IgLON5 antibodies. When the antibody status is unknown, a “probable” diagnosis requires neuropathological findings along with a compatible clinical history or confirmation of possession of HLA-DRB1*1001 and HLA-DQB1*0501 alleles. A “possible” diagnosis should be considered in cases with compatible neuropathology but without information about a relevant clinical presentation and immunological status. These criteria should help to identify undiagnosed cases among archival tissue, and will assist future clinicopathological studies of this novel disorder.

**Electronic supplementary material:**

The online version of this article (doi:10.1007/s00401-016-1591-8) contains supplementary material, which is available to authorized users.

## Introduction

An intriguing progressive neurological disorder at the crossroads of autoimmunity and neurodegeneration was recently described in eight patients affected by a previously unknown disorder characterized by non-REM sleep (NREM) parasomnias, sleep apnea, and stridor [16]. All patients had serum and CSF antibodies, mainly of the IgG4 subclass, against IgLON5, a neuronal cell-adhesion molecule with unknown function. In addition, all patients had the same HLA-DRB1*1001 and HLA-DQB1*0501 haplotype, which is very rare in the normal population [16]. Although these findings suggested an autoimmune disease, the symptoms did not respond to immunotherapy. Moreover, post-mortem findings in two patients suggested a novel tauopathy restricted to neurons and predominantly involving the hypothalamus and tegmentum of the brainstem without evidence of inflammation [16]. Since then, eight additional cases have been identified (four of them reported) based on the combined recognition of the characteristic sleep disorder, presence of IgLON5 autoantibodies and, when available, demonstration of the same haplotypes described above [2, 7, 14, 17].

While these clinical, immunological, and genetic features were being established, results of the post-mortem neuropathological examination of a third patient became available confirming the initial findings of a possible novel tauopathy. Therefore, a Task Force was organized to describe in detail the neuropathological features of the disorder with the aim of developing neuropathological diagnostic criteria which should also help to identify this disease in retrospective studies of archival tissue from patients whose diagnosis was not obtained during life.

## Materials and methods

The present study includes the neuropathological study of the brain of six patients. It comprises three previously reported cases, two of them from the archives of the IDIBAPS Neurological Tissue Bank, Barcelona, Spain [16] and one who had been identified and followed at the Department of Neurology, Medical University of Innsbruck, and underwent post-mortem examination at the Institute of Neurology, University of Vienna, Austria [7]. These three initial cases were comprehensively reviewed and a consensus regarding a preliminary set of neuropathological criteria was established. Based on these criteria two additional patients from Queen Square Brain Bank and the Division of Neuropathology, UCL Institute of Neurology, University College London, UK and a third one from the Institute of Pathology of the University of Ljubljana, Slovenia, were identified. Clinicopathological data of two of these patients who had died several years before the discovery of the anti-IgLON5 syndrome, were described as single case reports [13, 15].

In order to establish the neuropathological diagnostic criteria a meeting was held by four neuropathologists (EG, RH, GGK, TR), who were familiar with all of the six cases. Using a multi-header microscope the cases were re-evaluated and the histological features required for the neuropathological diagnosis of this novel tauopathy were agreed on by consensus. Subsequently, these neuropathological criteria along with clinical, immunological, and HLA haplotype findings were used by all the investigators [neuropathologists and clinicians (JD, FG, JS, BH)] to define three diagnostic levels of disease: “possible”, “probable”, and “definite”.

All six brains were processed according to standard neuropathological procedures. Formalin-fixed and paraffin-embedded tissue blocks representing frontal, temporal, parietal and occipital cortices, caudate, lenticular nucleus, hypothalamus, thalamus, hippocampus, parahippocampal gyrus, amygdala, midbrain, pons, medulla oblongata, spinal cord, and cerebellum were retrieved from the archives of the four centers and were cut as 5 μm thick sections. Sections were stained with haematoxylin and eosin method and in selected cases and areas tissue sections were also stained with Bielschowsky’s or Gallyas silver impregnation and Luxol-fast-blue/cresyl violet method. For immunohistochemistry a panel of primary antibodies was used in each laboratory (Table 1). Cases 1 and 2 were processed in Barcelona, case 3 in Vienna, case 4 originally in Ljubljana and later in Vienna, and cases 5 and 6 in Preston and London.

**Table 1:** Antibodies used for neuropathological characterization

Name	Clonality/clone	Company	Dilution
Anti-βA4-amyloid	mc, clone 6F/3D	DAKO, Glostrup, Denmark	1:400
Anti-phosphorylated tau	mc, clone AT8, pS202/pT205	Thermo Scientific, Rockford, IL, USA	1:200
Anti-ubiquitin	pc	DAKO, Glostrup, Denmark	1:400
Anti-alpha-synuclein	mc, clone KM51	Novocastra, Newcastle, UK	1:500
Anti-TDP-43	mc, clone 2E2-D3	Abnova, Taipei, Taiwan	1:500
Anti-RD3 tau	mc, clone 8E6/C11	Millipore, Temecula, CA, USA	1:1000
Anti-RD4 tau	mc, clone 1E1/A6	Millipore, Temecula, CA, USA	1:50
Anti-alpha-internexin	mc, clone 2E3	Invitrogen, CA, USA	1:800
Anti-p62	mc, clone 3/p62 lck ligand	BD Transduction Laboratories TM, NJ, USA	1:500

*mc* monoclonal, *pc* polyclonal

## Results

The main clinical and immunological characteristics of the six patients are summarized in Table 2. Three patients were male and the median age at the onset of symptoms was 53 years (range 48–77 years). The three patients diagnosed in life with positive IgLON5 antibodies were studied with video-polysomnography and presented a unique temporal sequence of sleep stages and behaviors, from very abnormal at the beginning of the night to close to normal by the end. The initiation and re-entering of sleep after awakening were characterized by undifferentiated non-rapid eye movement (NREM) sleep with frequent vocalizations, stereotyped movements, and finalistic behaviors (parasomnias). The REM sleep was present but only in the form of REM sleep behavior disorder. In addition, most patients had a sleep breathing disorder characterized by stridor and obstructive sleep apnea.

**Table 2:** Clinical features of the six patients

Patient #	1 (case 2^16^)	2 (case 7^16^)	3^7^	4^15^	5	6^13^
Gender	Male	Female	Female	Female	Male	Male
Age (years) at onset	53	76	54	77	48	49
Presenting symptoms	Sleep disorder	Sleep disorder	Sleep disorder	Dysphagia, dyspnea,	Vertigo, abnormal movements	Dysphagia, dyspnea
Disease duration	6 years	6 months	13 years	10 years	12 years	10 years
Excessive day sleepiness	Mild	No	Yes	No	Yes	Yes
Parasomnia	Yes (V-PSG)	Yes (V-PSG)	Yes (V-PSG)	Unknown	Yes (clinical report)	Unknown
Sleep apnea	Yes (V-PSG)	Yes (V-PSG)	Yes (V-PSG)	Unknown	Unknown	Yes
Stridor	Yes	Yes	Yes (endoscopy)	Yes (endoscopy)	Unknown	Unknown
Respiratory insufficiency	Yes	Yes (needed tracheostomy)	Yes (needed tracheostomy)	Yes (needed tracheostomy)	Yes (multiple ICU admissions)	Yes
Cognitive deterioration	No	No	No	No	No	No
Gait instability	Yes	Yes	Yes	No	Yes	No
Chorea	No	No	No	No	Yes	No
Parkinsonism	No	No	No	No	No	No
Limb ataxia	No	No	No	No	Yes	No
Ocular movements	Normal	Saccadic intrusions on pursuit	Normal (bilateral ptosis)	Horizontal nystagmus	Horizontal nystagmus, restricted up and lateral gaze	Normal (bilateral ptosis and horizontal nystagmus)
Bulbar symptoms	Dysphagia	Dysphagia, dysarthria	Dysphagia, dysarthria	Dysphagia	Dysphagia (gastrostomy feeding), dysarthria	Dysphagia (gastrostomy feeding)
Dysautonomia	Enuresis	No	No	No	Hypersalivation	No
Outcome	Sudden death during sleep	Sudden death during sleep	Death of respiratory failure	Sudden death during sleep	Died of respiratory arrest during hospital admission	Death of respiratory failure at home
IgLON5 antibodies	Positive	Positive	Positive	Not done	Not done	Not done
Diagnostic level^a^	Definite	Definite	Definite	Probable	Probable	Probable

*V-PSG* video-polysomnography

^a^See Table 4

The clinical history of the three patients in whom IgLON5 antibodies were not tested was dominated by bulbar dysfunction and repetitive episodes of respiratory insufficiency that required tracheostomy or multiple admissions to ICU. Other symptoms included gait instability, frequent falls, dysphagia, gaze palsies, central hypoventilation, dysautonomia, and chorea. These symptoms can suggest other diagnoses such as progressive supranuclear palsy or multiple system atrophy, although no parkinsonian signs were present. In none of these patients was the sleep formally studied, but in two of the cases symptoms of excessive daytime sleepiness, stridor, sleep apnea or parasomnia were documented (Table 2).

Table 3 summarizes the neuropathological features of the six cases. The detailed neuropathological reports of the two cases not previously reported and the re-evaluation of the previously published UK case [13] are presented below.

**Table 3:** Neuropathological characteristics including topographical distribution of hyperphosphorylated tau pathology

	Case 1 (case 2^16^)	Case 2 (case 7^16^)	Case 3	Case 4^15^	Case 5	Case 6^13^
Brain weight (in g)^a^	1410	1180	1290	1220	1422	1415
Macroscopic findings	Diffuse oedema	Mild prefrontal atrophy	Mild brainstem atrophy	Unremarkable	Unremarkable	Unremarkable
Brain region	pTau (AT8) pathology (RD3/RD4 isoforms in all cases)					
Frontal cortex	0	0	Isolated	Isolated	Isolated	Isolated
Temporal cortex	0	0	Isolated	Isolated	Isolated	Isolated
Parietal cortex	0	0	Isolated	N/A	N/A	0
Occipital cortex	0	0	Isolated	N/A	N/A	0
Ant cingulate cortex	Isolated	Isolated	Isolated	Isolated	Isolated	Isolated
Hippocampus CA4	++	+	++	+	++	++
Hippocampus CA2	+++	Isolated	+++	+++	+++	+++
Hippocampus CA1	++	+	++	++	++	++
Dentate gyrus	++	Isolated	++	+	+++	+++
Entorhinal cortex	++	+	++	+	++	++
Transentorhinal	Isolated	Isolated	++	Isolated	Isolated	Isolated
Amygdala	Isolated	+	+	++	+	Isol
n. basalis Meynert	++	+	++	+	N/A	+
Substantia innominata	++	++	+++	+++	N/A	N/A
Septal nuclei	++	Isolated	++	++	N/A	N/A
Striatum	0	Isolated	Isolated	Isolated	+	0
Pallidum, external	Isolated	Isolated	Isolated	Isolated	+	Isolated
Pallidum, internal	+	+	+	+	++	Isolated
Thalamus	Isolated	Isolated	Isolated	Isolated	+	Isolated
Zona incerta	Isolated	++	+++	+++	++	+
Subthalamic nucleus	Isolated	+	+	Isolated	+	+
Hypothalamus	++	++	+++	+++	+++	N/A
n. paraventricularis	+	+	+	+	N/A	N/A
n. supraopticus	+	+	+	+	N/A	N/A
n. dorsomedialis	+++	+	+++	+++	N/A	N/A
n. ventromedialis	+++	++	+++	+++	N/A	N/A
n. tuberales	++	++	++	++	N/A	N/A
n. posterior	+	++	+++	+++	N/A	N/A
Corpus mamillare	0	Isolated	Isolated	Isolated	N/A	N/A
Brainstem						
Laterodorsal tegmental n.	++	+++	+++	+++	+++	++
Pedunculopontine n.	++	+++	+++	+++	+++	+++
Periaqueductal gray	+	++	+++	+++	+++	+++
Substantia nigra	Isolated	Isolated	+	+	++	+
Locus coeruleus	+	+	++	++	++	+
Central raphe (pons)	++	+	+++	+	++	++
n. propii basis pontis	+++^b^	0	Isolated	0	+++ ^b^	Isolated
Dorsal n. vagal nerve	+	+	+++	+++	++	++
n. ambiguus	++	+++	+++	+++	+++	++
Gigantocellular nuclei	+++	+++	+++	+++	+++	+++
Inferior olives	Isolated	Isolated	Isolated	Isolated	+	Isolated
Cerebellum—cortex	++	0	+	Isolated	+	Isolated
Dentate nucleus	Isolated	Isolated	Isolated	Isolated	+	Isolated
Cervical spinal cord	+	+++	+++	+++	N/A	++

^a^Cases 1 and 2 unfixed; 3–6 fixed; ^b^ pretangles

### Case 3 (Vienna–Innsbruck, Austria)

**Fig. 1:**
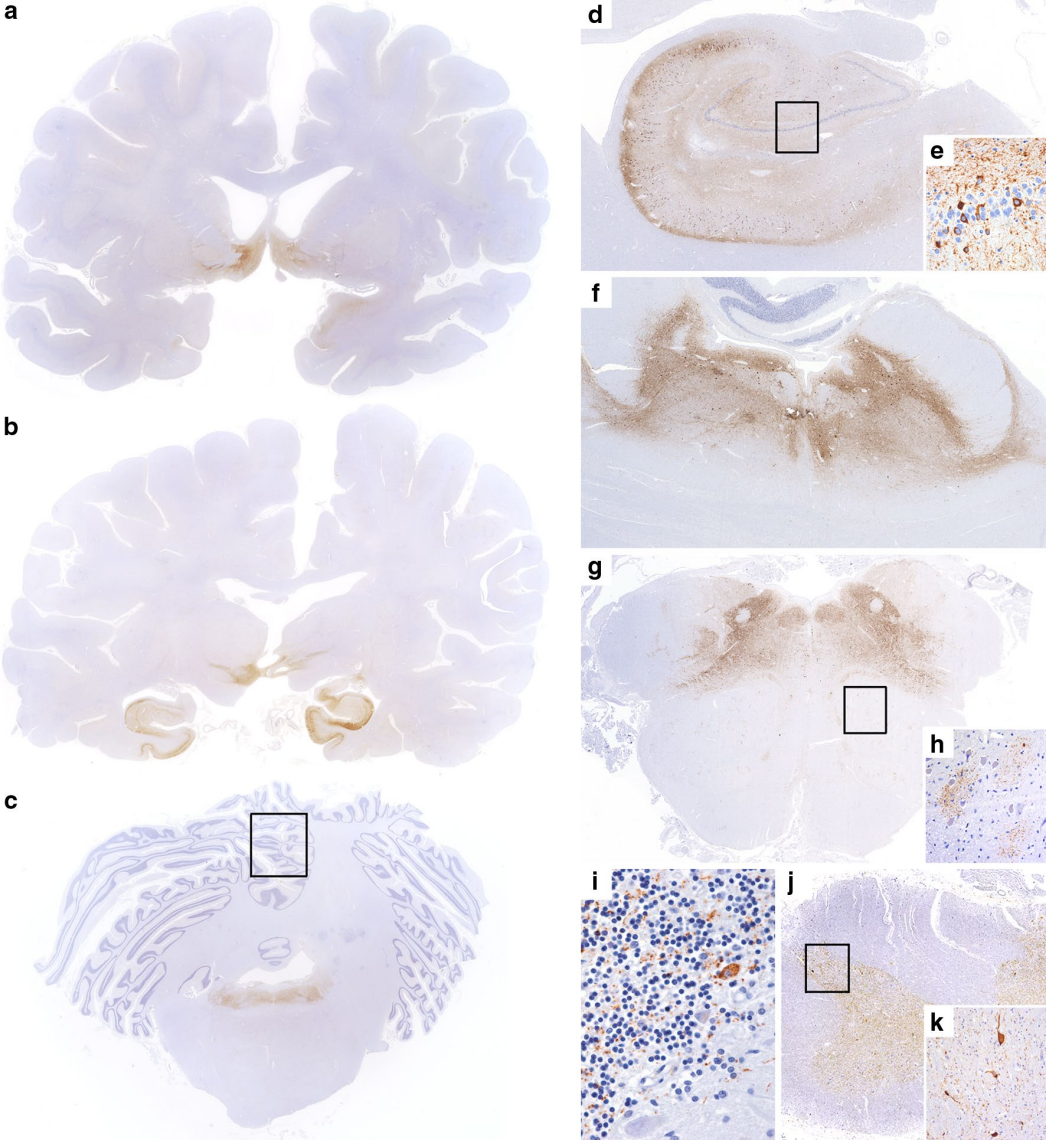
Distribution of tau related pathology in case 3. Coronal sections through the substantia innominata and hypothalamus (**a**), thalamus with nucleus ruber and substantia nigra (**b**), cerebellum with pons (**c**), higher magnification of hippocampus (**d**, **e**), pons (**f**), medulla oblongata at the level of olivary nucleus (**g**, **h**), cerebellar cortex (**i**), and cervical spinal cord (**j**, **k**). The tau pathology predominantly affects the hypothalamus, substantia innominata (**a**), zona incerta, hippocampus (**b**), and the tegmentum of the brain stem **(c)**; Tangles and threads are abundantly present in the pyramidal layer with the highest density in the CA2 sector (**d**). Pretangles are also present in the dentate gyrus (*rectangle* in **d** enlarged in **e**). High densities of tangles and threads are present in the tegmentum of pons (**f**) and medulla oblongata (**g**). Bush-like delicate processes accumulating around neurons are visible in the olivary nucleus (*rectangle* in **g** enlarged in **h**). Grain-like processes are mainly found in the vermis of the cerebellar cortex, occasionally, a few Purkinje cells show a cytoplasmic tau immunoreactivity (*rectangle* in **c** enlarged in **i**). Moderate tau pathology is apparent mainly in the dorsal horn of the spinal cord (**j**, enlarged in **k**)

**Fig. 2:**
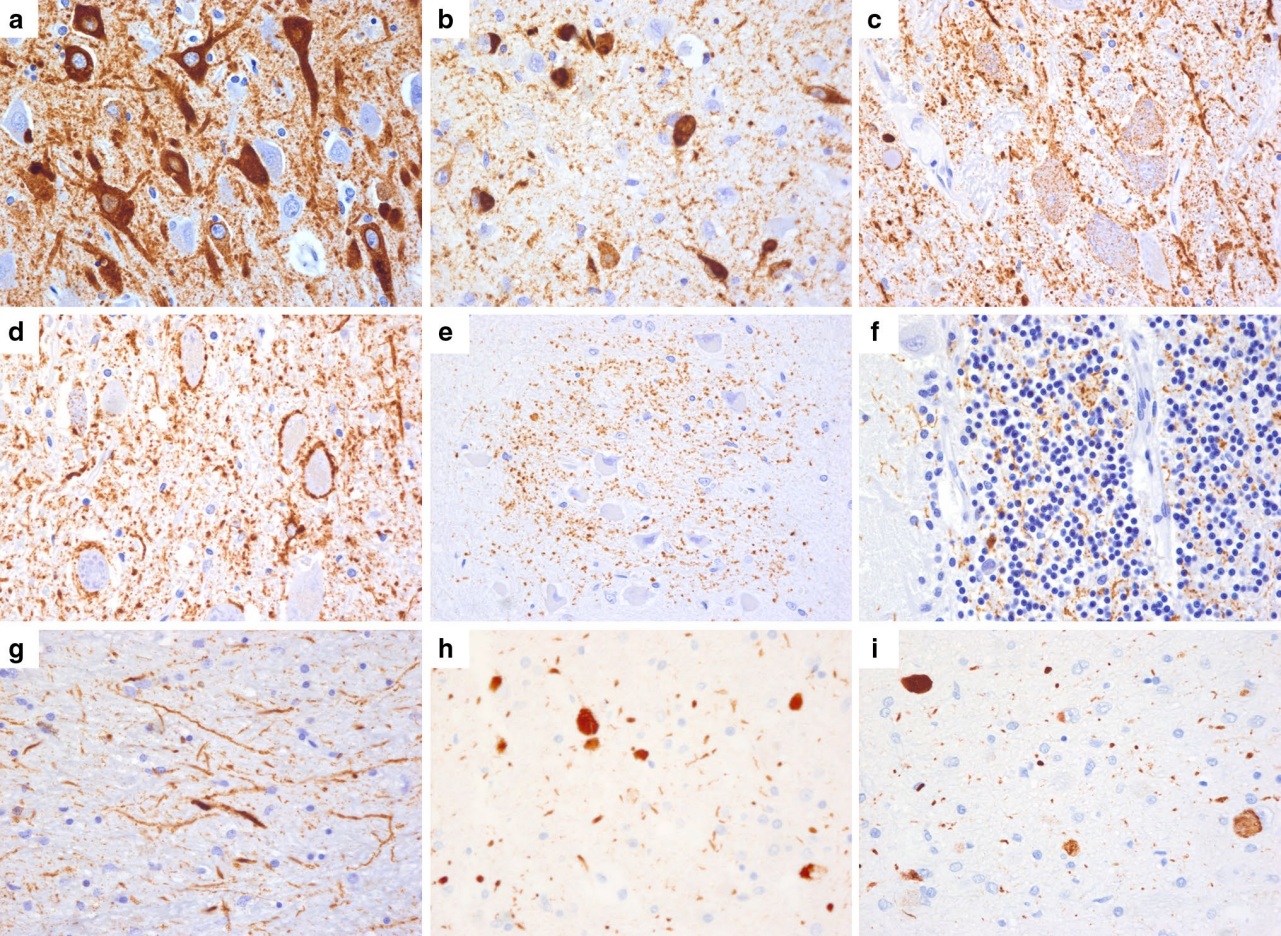
Morphology of tau related pathology in case 3. pTau related pathologies include NFT and pretangles (**a** CA2 sector of the hippocampus; **b** substantia innominata), diffuse fine granular cytoplasmic immunoreactivity (**c** gigantocellular nucleus of reticular formation), and numerous somato-synaptic immunoreactivity in the brain stem nuclei (**d** hypoglossal nucleus), bush-like delicate processes accumulating around neurons (**e** olivary nucleus), fine granular synaptic-like-deposits (**f** cerebellar cortex) and long coarse and fine threads (**g** substantia innominata). These immunomorphologies stain positive for three-repeat- (**h**) and four-repeat (**i**) tau isoforms. **a**–**g**: AT8 ×400; **h**: 3RT ×400; **i**: 4RT ×400

Detailed clinical information of the disease course has been previously reported [7] and is summarized in Table 2. Post-mortem examination of the cerebrum was performed after obtaining informed consent from the next of kin. Gross examination showed mild atrophy of superior cerebellar peduncles and brainstem tegmentum. Histology revealed a prominent tauopathy, characterized by numerous Gallyas-positive neurofibrillary tangles (NFT), diffuse granular cytoplasmic phospho-tau (pTau) immunoreacitivity (pretangles), and neuropil threads involving predominantly the hypothalamus, zona incerta, hippocampus, tegmentum of brainstem (mesencephalon, pons and medulla), and cervical spinal cord (Table 3; Fig. 1) in a symmetrical fashion. The hippocampus showed a high density of NFT and pretangles in the pyramidal cell layer (Fig. 2a), accentuated in the CA2 and CA4 sectors, and moderate amounts of pretangles in the dentate gyrus. Only occasional ghost tangles were detected. The pathology in the midbrain, pons, and medulla oblongata was mainly restricted to the tegmentum including periaqueductal gray matter, laterodorsal tegmental nucleus, pedunculopontine nuclei, median raphe nuclei, dorsal motor nucleus of the vagus nerve, nucleus ambiguus and neurons of the magnocellular nucleus (Fig. 2c). Substantia nigra was only mildly affected, and the subthalamic nucleus and basis pontis showed isolated threads and pretangles (Fig. 2d). The inferior olivary nucleus was well preserved but had frequent clusters of fine threads surrounding individual neurons (Fig. 2e). The cerebellar cortex was globally well preserved, but showed a fine granular synaptic-like pTau immunoreactivity pattern involving the glomerula of the granular cell layer of the vermis (Fig. 2f). In addition, a few Purkinje cells showed diffuse cytoplasmic pTau positivity. There was a prominent involvement of the cervical spinal cord with marked pTau pathology in the dorsal horns and lesser involvement of the anterior horns (Fig. 1j), with decreasing intensity further caudally in the lumbar spinal cord, where pathology became less evident but was still visible in the dorsal horn. pTau immunoreactivity was almost entirely confined to neurons and the tau-positive structures were also labeled with both, the 3R-tau (Fig. 2h) and 4R-tau (Fig. 2i) antibodies. All these areas showed moderate microglial activation. Neuronal loss correlated with the presence of NFT. The cranial nerves were not affected. Only very occasional coiled bodies and tau-positive granular fuzzy astrocytes were visible in the hypothalamus, substantia nigra, and anterior horn of the spinal cord. Other morphological alterations included a few ballooned neurons in the amygdala. Anti-LCA revealed only a few meningeal and perivascular leukocytes mainly composed of CD3 and CD8 positive T cells and a few CD20 and CD79A B cells in the hypothalamus, brainstem, and cerebellum without any parenchymal involvement that could suggest an inflammatory process. No obvious IgG4 deposits were detected, except for a faint staining of the cerebellar glomerula of the vermis (data not shown). Complement deposits were not visible.

### Case 5 (London, UK)

A 48-year-old man developed recurrent vertigo and marked involuntary fidgety movements. At the age of 53 he developed breathing and swallowing difficulties. Multiple episodes of aspiration pneumonia and respiratory arrests warranted four admissions to the intensive care unit and, on two occasions, intubation for ventilator support was required. Due to recurrent aspiration, a gastrostomy tube was inserted for feeding. One year later, he developed progressive difficulties with his balance and intermittent diplopia. He had lateral and upgaze nystagmus with limitation of vertical and abduction eye movements. There was cerebellar dysarthria, finger-nose dysmetria, and he was unable to tandem walk. Choreiform movements were present in all limbs. Deterioration in excessive daytime somnolence caused him to fall asleep even when he was standing. His wife reported that his breathing pattern changed as soon as he was asleep but no abnormal movements were noted. During a hospital admission to investigate his sleep attacks, he had a respiratory arrest and died at the age of 60.

Neuropathological examination was performed after appropriate written informed consent from the next of kin had been obtained. Consent for the use of this case for research purposes was also given. The brain was macroscopically unremarkable. Microscopic studies showed an increase in number of reactive astrocytes in the striatum and globus pallidus. There was some loss of neurons with accompanying astrogliosis in the dorsolateral aspect of the subthalamic nucleus while the substantia nigra was well populated with pigmented and non-pigmented neurons. There was some reduction of the height of the pontine tegmentum, which was marked in the medulla oblongata. Tau immunohistochemistry using the AT8 antibody demonstrated only occasional NFT and pretangles in neocortical areas. All hippocampal subregions were affected by neuronal tau pathology; there were moderate numbers of pretangles and NFT in the CA1 hippocampal subregion, which were sparse in the CA3 and CA4 subregions. There were frequent pretangles in CA2 and also in the granule cells of the dentate gyrus, which were ubiquitin and p62-negative. Fine neuropil threads were present in all hippocampal subregions. The tau pathology was similar, but mild in the subiculum while moderate numbers of Gallyas-positive NFT and neuropil threads were seen in the entorhinal cortex. There were scattered tau-positive neuropil threads, pretangles and occasional NFT in the striatum. Similar, but slightly more severe pTau pathology was seen in the globus pallidus. The subthalamic nucleus contained moderate numbers of pretangles, NFT and neuropil threads. A conspicuous feature was the severe tau deposition in the posterior hypothalamus and brainstem structures. In the midbrain a significant proportion of the substantia nigra pars compacta neurons showed granular or dot-like tau positivity, which was also seen in neuronal processes. Similar tau pathology was seen in the midbrain tegmentum, periaqueductal gray matter and tectum. The majority of the neurons of the otherwise well-populated locus coeruleus contained similar neuronal tau-positive inclusions and the pontine tegmentum and base were also extensively affected by similar tau pathology. The tau deposition was particularly severe in the tegmentum of the medulla including the dorsal motor nucleus of the vagus nerve, nucleus tractus solitarius, nucleus ambiguus and neurons of the magnocellular nucleus were severely affected. In the inferior olive neurons were often surrounded by a meshwork of fine tau-positive processes and dot-like tau positivity. There were scattered pretangles and NFT, neuropil threads and clusters of fine tau-positive dots in the cerebellar dentate nucleus. There was rather widespread granular tau positivity in the cerebellar cortex and an occasional Purkinje cell showed fine, dot-like tau positivity. Differential tau immunohistochemistry confirmed that the pTau deposits were composed of both 3R-tau and 4R-tau isoforms.

### Case 6 (London, UK)

For clinical history and the distribution of the NFT pathology see Lidov et al. [13]. Tau immunohistochemistry with the AT8 antibody was carried out using tissue sections of paraffin blocks available in the archives of the Division of Neuropathology, UCL Institute of Neurology. The distribution of the pTau pathology, including involvement of the hippocampus was rather similar to that described in case 5. However, there were some differences, which included that in this case the striatum was virtually devoid of tau pathology and the involvement of the globus pallidus and subthalamic nucleus was considerably milder than that observed in case 5. The brainstem tegmentum showed severe deposition of disease-associated tau in a distribution as described above, but the substantia nigra and pontine base showed only sparse tau-positive pretangles, NFT and neuropil threads. There was tau pathology of moderate degree in the posterior horns of the cervical and thoracic cord (these were the levels that were available for review) and sparse neuropil threads and occasional NFT were also seen in the anterior horns. Occasional tau-positive granular deposits were found in the cerebellar cortex. The tau inclusions were both 3R-tau and 4R-tau immunoreactive.

### Summary of neuropathological features and suggested neuropathological criteria

**Fig. 3:**
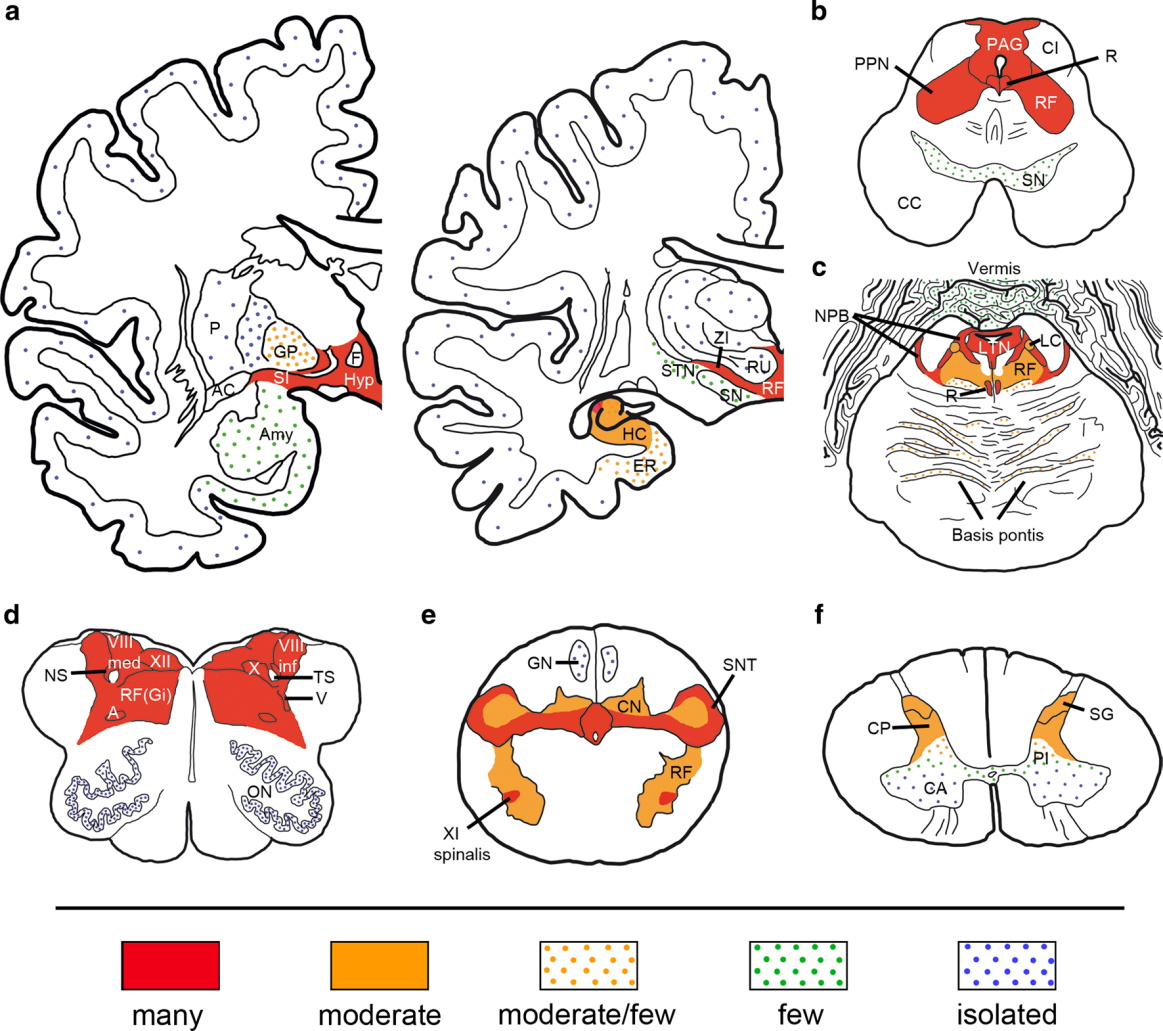
Schematic distribution of tau pathology in anti-IgLON5 related tauopathy. Coronal sections through the amygdala and the lateral geniculate body (**a**), midbrain (**b**), pons (**c**), medulla oblongata [level of olivary nucleus (**d**) and decussation of pyramids (**e**)], and cervical spinal cord (**f**). Scoring of the frequency of tau pathology in sections stained with tau AT8 is based on the number of tangles and threads: *red* many; *orange* moderate; *orange dots* moderate/few; *green dots* few; and *blue dots* isolated. *A* nucleus ambiguus, *AC* anterior commissure, *Amy* amygdala, *CA* anterior horn, *CC* crus cerebri, *CI* inferior colliculus, *CN* cuneate nucleus, *CP* posterior horn, *ER* entorhinal cortex, *F* fornix, *GP* pallidum, *GN* gracile nucleus, *HC* hippocampus, *Hyp* hypothalamus, *LC* locus coeruleus, *LTN* laterodorsal tegmental nucleus, *NPB* parabrachial nuclei, *NS* solitary nucleus, *ON* olivary nucleus, *P* putamen, *PAG* periaquaeductal gray, *PI* pars intermedia, *PPN* pedunculopontine nucleus, *RU* nucleus ruber, *R* raphe nucleus, *RF* reticular formation, *RF(Gi)* gigantocellular reticular nucleus, *SG* substantia gelatinosa, *SI* substantia innominata, *SN* substantia nigra, *SNT* nucleus of spinal tract of trigeminal nerve, *STN* subthalamic nucleus, *TS* solitary tract, *V* trigeminal nucleus, *VIII inf* inferior vestibularis nucleus, *VIII med* medial vestibularis nucleus, *X* dorsal nucleus vagal nerve, *XI spinalis* spinal accessory nucleus, *XII* hypoglossal nucleus, *ZI* zona incerta

The neuropathological findings in the six patients showed remarkable similarity consistent with a neurodegenerative disease with neuronal loss and gliosis, without the presence of inflammatory infiltrates. The most relevant finding was a prominent neuronal accumulation of pTau, comprising both 3R-tau and 4R-tau isoforms, involving the hypothalamus and more severely the tegmental brainstem nuclei, with a cranio-caudal gradient of severity, reaching the upper cervical cord. The cerebral cortex, basal ganglia, thalamus and subthalamic nucleus were mostly unaffected or mildly affected. In five cases the entorhinal cortex and hippocampus were also affected including dentate gyrus and CA4 to CA1 sectors, with variable involvement of transentorhinal region. Interestingly, CA2 sector was consistently involved. There was a lack of glial tau pathology except for the presence of isolated coiled bodies and granular fuzzy astrocytes in the hypothalamus and amygdala in cases 3 and 4. A detailed semiquantitative assessment of pTau pathology in different brain areas of all cases is represented in Table 3 and Fig. 3 shows a schematic heat-map representation of the distribution and severity of pTau pathology throughout the brain for all cases.

There were no other abnormal deposits of proteins, or if present these were mild and localized in a few areas suggesting the co-existence of other neurodegenerative or age-related pathologies. Case 2 showed isolated Bunina bodies and TDP43 protein aggregates in few brainstem and motor neurons in the spinal cord. A few neuritic plaques were observed in cases 2, 4, and 6, along with cerebral amyloid angiopathy in cases 2 and 4. Case 4 additionally showed scattered alpha-synuclein positive Lewy bodies and Lewy neurites in the substantia nigra and dorsal nucleus of the vagus nerve and argyrophilic grain pathology, and case 5 showed sparse neuronal cytoplasmic TDP43 protein inclusions in the granule cells of the dentate gyrus.

Based on the similarities of these cases we propose research criteria for the neuropathological diagnosis of the tauopathy underlying the anti-IgLON5 syndrome (Table 4) and recommend a protocol of tissue sampling (Table 5). When a neuropathologist is confronted with a case with such a neuropathological phenotype in conjunction with positive IgLON5 antibodies in CSF or serum, the diagnosis of “*definite anti*-*IgLON5*-*related tauopathy*” should be considered. In contrast, when the information of the anti-IgLON5 antibodies status is missing but the clinical history is compatible or the patient harbored the HLA-DRB1*1001 and HLA-DQB1*0501 alleles, then the diagnostic category of “*probable anti*-*IgLON5*-*related tauopathy*” should be applied. The category of “*possible anti*-*IgLON5*-*related tauopathy*” should be reserved for those cases where clinical data are not available (e.g. due to the retrospective identification of archival cases or other reasons) and ancillary tests (anti-IgLON5 antibodies and/or HLA-DRB1*1001 and HLA-DQB1*0501 alleles) could not be performed. Note that both the HLA genotyping and the anti-IgLON5 antibody assay could be done post-mortem from DNA samples and serum or ventricular CSF, respectively. No exclusion criteria have been proposed as the whole spectrum of pathological changes and possible concomitant pathologies are yet unknown.

**Table 4:** Proposed neuropathological criteria to define the tauopathy underlying the anti-IgLON5 syndrome

Possible
All of the following requirements
Neurodegenerative features with neuronal loss and gliosis in brain areas showing hyperphosphorylated (p)Tau pathology without the presence of inflammatory infiltrates
Selective neuronal involvement by deposition of pTau in the form NFT, pretangles and neuropil threads with both 3R-tau and 4R-tau isoforms contributing to the inclusions
The pTau pathology predominantly affects subcortical structures^a^, including the hypothalamus, brainstem tegmentum and upper spinal cord
Probable
Criteria of “possible” AND at least one of the following
Clinical history suggestive of a sleep disorder (NREM and REM parasomnia with sleep apnea), or brainstem, mainly bulbar dysfunction^b^
Presence of HLA-DRB1*1001 and HLA-DQB1*0501 alleles
Definite
Criteria for “*possible*” AND presence of IgLON5 antibodies in CSF or serum^c^

^a^Hippocampus generally involved, except for one patient

^b^Includes dysarthria, dysphagia, central hypoventilation, stridor

^c^IgLON5 positivity is detected by cell-based assay in serum at 1/40 and in CSF at 1/2

**Table 5:** Recommended minimal sampling protocol for the diagnosis of anti-IgLON5 related tauopathy

1	Frontal cortex (e.g., middle frontal gyrus)
2	Temporal cortex (e.g., middle temporal gyrus)
3	Pre/postcentral gyri
4	Parietal cortex (e.g., inferior parietal gyrus)
5	Occipital cortex including striate and parastriate cortex
6	Posterior hippocampus at the level of the lateral geniculate body
7	Amygdala
8	Striatum at the level of the nucleus accumbens
9	Basal ganglia at the level of the anterior commissure to include the nucleus basalis and diagonal band
10	Anterior and posterior hypothalamus
11	Anterior, dorsomedial, ventrolateral and posterior thalamus
12	Subthalamus
13	Midbrain at the level of the emergence of the 3rd cranial nerve
14	Pons at the level of the locus coeruleus
15	Medulla at the level of the caudal end of the 4th ventricle to include the 12th nerve nucleus and inferior olive
16	Cerebellar cortex, white matter and dentate nucleus
17	Spinal cord (at least cervical level)
18	Blood and CSF for antibody and HLA testing

## Discussion

We report here the main neuropathological features underlying the anti-IgLON5 syndrome that are different from those of other tauopathies [4, 11]. The three main findings are (1) the subcortical topographical distribution of the tau pathology with predominant involvement of the hypothalamus and brainstem tegmentum, with a rostro-caudal gradient of severity to include the upper cervical cord, (2) the nearly exclusive neuronal tau pathology with little or no involvement of glia and the white matter, and (3) composition of disease-associated tau of both 3R-tau and 4R-tau isoforms.

We propose that the tauopathy underlying the anti-IgLON5 syndrome can be diagnosed with three levels of probability: definite, probable, and possible. Accordingly, for example the previously reported case by Kaphan et al. [10] would fulfill criteria of “probable” anti-IgLON5-related tauopathy as the neuropathological findings and clinical features are supportive of the diagnosis. We believe that these neuropathological features present in our six cases make this entity unique and the tau pathology seen did not meet the diagnostic criteria of any of the known primary tauopathies. In our cases the neuronal tau pathology showed a characteristic distribution with preferential involvement of the hypothalamus, tegmentum of the midbrain, pons, and medulla oblongata, which affected the periaqueductal gray matter, laterodorsal tegmental nucleus, pedunculopontine nucleus, median raphe nucleus, dorsal motor nucleus of the vagus, nucleus ambiguus, and the magnocellular nuclei of reticular formation with only mild or no involvement of substantia nigra and inferior olivary nucleus. These areas have been implicated in sleep regulation and correlate well with clinical symptoms of the patients [5]. There is additional involvement of the spinal cord with neuronal accumulation of pTau in the dorsal horns, and to a lesser extent, in the anterior horns. The tau pathology also shows a cranio-caudal gradient in that the cervical spinal cord is more severely affected than the lumbar cord. Discrete fine granular tau immunoreactivity is also observed in glomerula of the granular cell layer of the cerebellar cortex in the vermis. In addition, a few Purkinje cells may show diffuse cytoplasmic pTau positivity.

This condition has distinct microscopic appearances allowing its differentiation from other ‘primary’ tauopathies, although the topographical distribution of the tau pathology is, to some extent, reminiscent of that seen in progressive supranuclear palsy (PSP). Indeed, one post-mortem case has been previously reported as “early” PSP [10] and another case clinically mimicking PSP and with serum—but not CSF—IgLON5 antibodies has been described [2]. However, the anti-IgLON5-related tauopathy can clearly be differentiated from PSP, even from its variants with more restricted tau pathology [4, 18] or other 4R tauopathies such as corticobasal degeneration (CBD) [4], globular glial tauopathies (GGT) or argyrophilic grain disease (AgD) by the morphological patterns of the tau pathology, including the absence of glial pathology (tufted astrocytes or coiled bodies), and sparse supratentorial and basal ganglia involvement [11]. A severe brainstem involvement clinically causing REM sleep behavior disorder, stridor, respiratory insufficiency and death during sleep, cerebellar dysfunction including nystagmus and limb/gait ataxia, and autonomic dysfunction is also seen in multiple system atrophy, which is associated with alpha-synuclein accumulation in glial cytoplasmic inclusions and less frequently neuronal cytoplasmic and nuclear inclusions [8].

As in Alzheimer’s disease, the tau filaments seen in the anti-IgLON5 syndrome ultrastructurally appear as paired helical filaments [13] and the differential tau immunohistochemistry confirms that they are composed of both 3R-tau and 4R-tau isoforms. In addition to Alzheimer’s disease, tau inclusions composed of both 3R-tau and 4R-tau isoforms occur also in several other disorders such as postencephalitic parkinsonism, chronic traumatic encephalopathy, Parkinson-dementia complex of Guam, and FTDP-17 due to MAPT mutations. However, these disorders frequently have glial involvement, show different topographical distribution of abnormalities, and present with different clinical syndromes [11].

In addition to the prominent brainstem involvement, five of the six patients had neuronal tau pathology in the hippocampus with frequent pretangles in CA4 and CA2 sector, and NFT in CA1 sector and entorhinal cortex, with variable involvement of transentorhinal region, and ring-shaped pretangles in the granule cells of the dentate gyrus. Selective involvement of CA2 sector has been reported to occur more frequently in 4R-tauopathies [9]. The tau distribution was however more similar to that described in primary age-related tauopathy (PART) [3] which is considered a common pathology associated with human aging. More neuropathological studies are needed to clarify whether the hippocampal involvement in our patients indicates the presence of an associated PART as an accelerated aging or is part of the neuropathological signature of anti-IgLON5-related tauopathy. The prominence of hippocampal pTau pathology and its extension beyond the boundaries of the medial temporal lobe in relatively young patients (mean age 68.2) argues for the latter possibility. The mild to moderate involvement of the locus coeruleus might also reflect an age-related process, since this has been reported as another site of early tau hyperphosphorylation and accumulation (pretangle stage) even before puberty or in early young adulthood [1]. Similarly, occasional coiled bodies and tau-positive fuzzy astrocytes observed in one case suggest the presence of a mild aging-related tau astrogliopathy (ARTAG) [12].

The most intriguing aspect of the disease is the association of neuropathological findings suggesting a non-inflammatory degenerative process with the presence of highly specific antibodies against IgLON5 (a neuronal cell-adhesion molecule) and a strong association with the HLA system. A possible mechanism that could explain tau hyperphosphorylation and accumulation in neurons might relate to one of the functions of IgLON family as glucosyl-phosphatidyl-inositol anchored proteins and its role in membrane stabilization [6]. An antibody-induced dysfunction of IgLON5 may disrupt the interaction of this protein with the internal cytoskeletal network, de-stabilize the neuronal microtubular system and induce hyperphosphorylation and accumulation of the microtubule-associated protein tau, leading to neuronal dysfunction and ultimately to neurodegeneration. The distribution of pTau pathology could raise the possibility of a trans-synaptic spread of tau from selectively vulnerable neuronal populations by neuronal connectivity, i.e. from several brainstem nuclei (e.g. dorsal raphe, reticular formation, locus coeruleus), caudally to posterior and intermediate spinal cord gray matter and rostrally to hypothalamic nuclei, although no overt involvement of other interconnected areas has been observed (e.g. amygdala, striatum, thalamus, neocortex) (supplementary Fig. 1).

However, the intriguing coexistence of neuropathological and immunological findings and the precise mechanism of the cross-talk between these two potential pathogenic mechanisms need to be elucidated.

In summary, since the publication of two cases of a novel, brainstem accentuated neuronal 3R and 4R tauopathy associated with IgLON5 autoantibodies in patients with apnea and NREM parasomnia, a new case has been confirmed in Austria and three additional cases, one from Slovenia and two from the UK have been retrospectively identified on the basis of their nearly identical neuropathological phenotype. We suggest including these cases under the umbrella term of a single clinicopathological entity termed “anti-IgLON5-related tauopathy”. To facilitate communication with clinicians and researchers and to provide a framework for future clinicopathological and research studies we propose here research criteria for the neuropathological diagnosis of this unique tauopathy and recommend a protocol for tissue sampling. This information could also be of value in the re-evaluation of archival cases. Clearly, the recognition of more cases will be of utmost importance to identify the entire spectrum of the clinicopathological presentation and to elucidate the role of IgLON5 antibodies in initiating a cellular cascade finally resulting in abnormal hyperphosphorylation, aggregation and accumulation of tau. Only a better understanding of this interplay can result in the design of appropriate treatment strategies that can modify disease progression and consequently mitigate the risk of sudden death during sleep.

## Electronic supplementary material

Below is the link to the electronic supplementary material.

**Supplementary Figure 1**: Simplified schematic representation of neuroanatomical pathways connecting different brainstem nuclei upwards and downwards by which a trans-synaptic propagation of IgLON5-related pathology could be postulated. Gray shaded boxes indicate nearly non-affected areas. (TIFF 23576 kb)
