# Supplementary figures and images for: Neuropathological criteria of anti-IgLON5-related tauopathy
Source: Acta Neuropathol. 2016 Jun 29;132(4):531–43. doi: 10.1007/s00401-016-1591-8 (PMC5023728; doi:10.1007/s00401-016-1591-8)

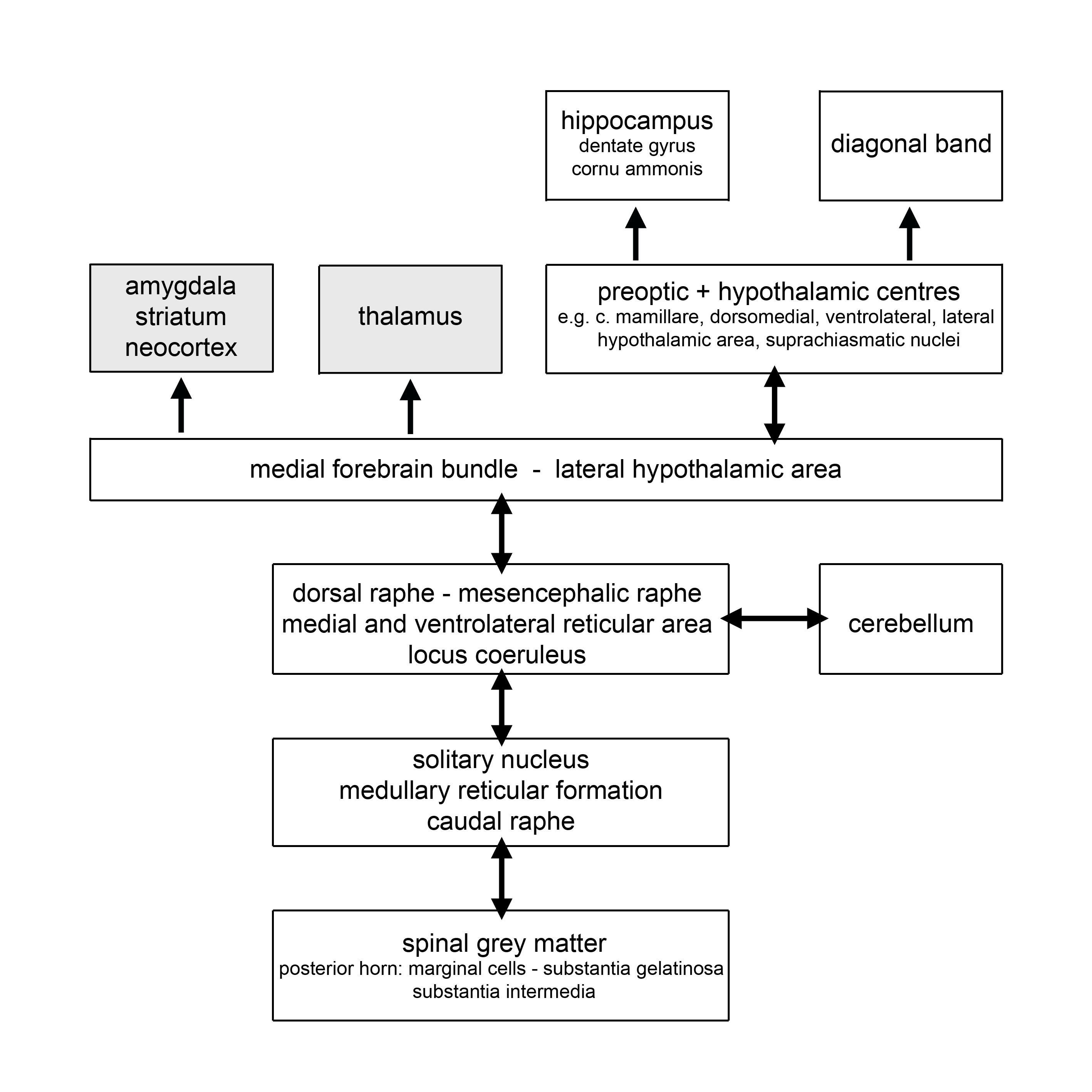

Supplement: Supplementary file 1 — Supplementary Figure 1: Simplified schematic representation of neuroanatomical pathways connecting different brainstem nuclei upwards and downwards by which a trans-synaptic propagation of IgLON5-related pathology could be postulated. Gray shaded boxes indicate nearly non-affected areas. (TIFF 23576 kb) [file 401_2016_1591_MOESM1_ESM.tif]
